# Efficacy of Traditional Chinese Exercise in Improving Gait and Balance in Cases of Parkinson's Disease: A Systematic Review and Meta-analysis

**DOI:** 10.3389/fnagi.2022.927315

**Published:** 2022-06-30

**Authors:** Minmin Wu, Qiang Tang, Linjing Wang, Mei Zhang, Wenjing Song, Lili Teng, Luwen Zhu

**Affiliations:** ^1^Department of Rehabilitation Medicine, Heilongjiang University of Chinese Medicine, Harbin, China; ^2^The Second Affiliated Hospital of Heilongjiang University of Chinese Medicine, Harbin, China

**Keywords:** traditional Chinese exercise, gait, balance, Parkinson's disease, meta-analysis

## Abstract

**Background:**

The efficacy of traditional Chinese exercise (TCE)-based intervention in the improvement of motor function in patients with Parkinson's disease (PD) is controversial. The present study aimed to assess the effects of TCE on balance and gait outcomes, as well as motor symptoms in individuals with PD, and evaluate potential discrete moderators such as TCE dosage-related variables.

**Method:**

PubMed, Embase, Cochrane's Library, Web of Science, Medline, and Scopus were systematically searched from their dates of inception to February 2022. All studies were randomized controlled trials (RCTs) of TCE-based interventions for PD. The treatment effects were estimated using a random-effect meta-analysis model with standardized mean differences (Hedges' g). The Physiotherapy Evidence Database was used to evaluate the methodological quality of the study.

**Result:**

Fifteen studies involving a total of 873 participants were included in the final analysis. The meta-analytic findings revealed significant improvements in balance outcomes [Berg Balance Scales (BBS) (*g* = 0.83, 95% CI = 0.37–1.29, *p* = 0.000, I^2^ = 84%), time up and go (TUG) (*g* = −0.80, 95% CI = −1.13– −0.47, *p* = 0.000, I^2^ = 81%), and the one legged blind balance test (*g* = 0.49, 95% CI = 0.13–0.86, *p* = 0.01, I^2^ = 10%)], as well as gait outcomes [gait velocity (*g* = 0.28, 95% CI = 0.02–0.54, *p* = 0.04, I^2^ = 64%), 6-min walking test (6MWT) (*g* = 0.32, 95% CI 0.01–0.62, *p* = 0.04, *I*^2^= 15%), stride length (*g* = 0.25, 95% CI = 0.08–0.41, *p* = 0.003, I^2^ = 42%)], and motor symptoms [Unified Parkinson's Disease Rating Scale part III (UPDRS-III) (*g* = −0.77, 95% CI = −1.06– −0.48, *p* = 0.000, I^2^ = 76%)]. However, cadence (*g* = −0.03) and step length (*g* = 0.02) did not differ significantly. The moderator shows that the effects of TCE on BBS and gait velocity were moderated by Pedro score, exercise type, control group type, and number of sessions. Meta-regression found that TCE (exercise duration, number of sessions, and session duration) was significantly associated with improved UPDRS-III and BBS scores.

**Conclusion:**

These findings provide evidence for the therapeutic benefits of TCE as an adjunct therapy for patients with PD. TEC dosage (high-intensity long sessions) may moderate some favorable effects.

**Systematic Review Registration:**

PROSPERO, identifier CRD42022314686.

## Introduction

Parkinson's disease (PD) is characterized by bradykinesia, balance disruption, rigidity, and gait impairment, and is the second most common neurodegenerative disease after Alzheimer's disease (Kalia and Lang, 2015; Abbruzzese et al., 2016). Recent research has revealed that the disease impacted ~6.1 million people worldwide in 2016, among whom more than 1% of people over 65 years of age were affected. It is expected to affect 13 million people by 2040 (Dorsey et al., 2018; Gomez-Inhiesto et al., 2020). The degradation of the nigrostriatal dopaminergic system is an extremely significant pathogenic alteration in PD (Kordower et al., 2013; Biondetti et al., 2021). There are no disease-modifying treatments for PD, and only dopaminergic replacement therapy combined with medication therapy and deep brain stimulation can improve symptoms (Connolly and Lang, 2014; Curtze et al., 2015). However, gait and balance problems persist in people with PD despite optimum medication, and commonly result in falls with potentially serious repercussions (van der Marck et al., 2014; Swanson and Robinson, 2020; Longhurst et al., 2022).

Experts recommend exercise and physical therapy as effective adjuvants to levodopa therapy since pharmaceutical and surgical management of PD remains inadequate (Fox et al., 2011). Physical activity slows the decline of motor capabilities and extends functional independence in people with PD (Earhart and Falvo, 2013; van der Kolk and King, 2013). The health advantages of alternative traditional Chinese exercise (TCE) such as Tai Chi and Qigong (Baduanjin) are becoming increasingly popular. Tai Chi and Qigong (Baduanjin) currently have the most substantial evidence of efficacy available, particularly in the improvement of muscle strength, aerobic capacity, and postural stability (Field, 2011; Carcelén-Fraile et al., 2021; Yuen et al., 2021). These are low-cost, mild-to-moderate-intensity workouts that emphasize physical–mental link training with slow, gentle, and symmetrical motions, a meditative state, and breathing control that must be linked with gradual body activity (Wang et al., 2015; Song et al., 2017; Zou et al., 2018a,b; Fidan et al., 2019).

According to a large body of research, TCE improves balance and gait function in patients with PD. Zhu et al., for example, found that 3-month Tai Chi training improves motor symptoms, balance, and cognitive function in patients with PD in randomized controlled research (Zhu et al., 2020). Dong et al. discovered that Baduanjin exercise helped to improve balance, gait, and daily activities in patients with PD (Dong et al., 2021). However, several systemic reviews have yielded inconsistent findings on the health advantages of TCE in individuals with PD. For example, a systemic review of five studies found that Tai Chi practice can reduce fall rates and enhance balance and functional mobility in patients with PD compared with no intervention or alternative physical training (Liu et al., 2019). Conversely, a systematic review conducted by Yang et al. revealed that Tai Chi exercise did not significantly affect gait velocity, step length, or gait endurance in patients with PD (Yang et al., 2014).

Furthermore, previous systematic reviews habitually focus on single exercises, which may not reflect TCE's overall health effects accurately. To date, no meta-analyses have been conducted to assess whether various types of TCE (Tai Chi and Qigong) and dosage variables (frequency, exercise duration, and number and duration of sessions) affect TCE-induced balance, gait, and motor symptoms. The effect of variables on the effect size of trials was not assessed using meta-regression analysis. Although some previous systematic reviews had small sample sizes, none of them used Hedges' g statistic to calculate the effect size. These disparate research findings suggest that additional, comprehensive studies are warranted to confirm the effects of TCE on gait and balance outcomes in people with PD.

Therefore, the present study was conducted with the first goal of determining the effects of TCE balance and gait outcomes, along with motor symptoms, in individuals with PD. The second goal was to determine whether any potential moderators (e.g., high and low methodological quality, active and non-active control groups) and a continuous meta-regressor for TCE dosage-related variables (e.g., frequency, exercise duration, and number and duration of sessions) that influenced the intervention effects were present.

## Methods

The meta-analysis adhered to the 'Preferred Reporting Items for Systematic Reviews and Meta-Analyses' criteria (PRISMA) (Moher et al., 2009).

### Search Strategy

A two-stage literature search was conducted to locate relevant articles. To start, electronic databases (PubMed, Embase, Cochrane's Library, Web of Science, Medline, and Scopus) were searched from the date of their establishment to February 2022. Second, reference lists of published publications were combed for research not indexed in electronic databases. The following keywords were used: (1) “Tai Chi” OR “Qigong” OR “Baduanqin” OR “Wuqinxi” OR “Yijinjing” OR “traditional Chinese exercise; AND (2) “PD”; AND (3) “randomized controlled trials” OR “clinical trial.” The Supplementary Material contains a complete description of the search approach.

### Eligibility Criteria

Studies were included if the following criteria were met: (1) the study was a randomized controlled trial (RCT); (2) the target population was individuals with PD; (3) type of intervention: an experimental group included in the form of TCE [Tai Chi, Qigong (Baduanjin)]; (4) TCE was compared with a control group exposed to any- (active) or no intervention (non-active); (5) balance function, gait parameters, and motor symptoms were evaluated as the outcomes and all included outcome indicators were assessed at the beginning and the end of the intervention; (6) studies written in English. The exclusion criteria were as follows: (1) non-RCT studies; (2) animal studies, case reports, conference abstracts, and letters to the editor; (3) insufficient data or irrelated outcomes; (4) non-English reviews.

### Study Selection

Two reviewers (MW and MZ) independently assessed the article's eligibility based on the title and abstract retrieved. The full text was reviewed if the abstract was considered relevant or ambiguous. Any unclear information was obtained *via* an e-mail to the corresponding author. If there was disagreement, an agreement was reached after a third reviewer evaluated the article.

### Data Extraction and Quality Assessment

Two reviewers (MW and MZ) assessed the articles and extracted data separately and independently. Details of the retrieved articles are summarized in Table 1. Data regarding the study characteristics (first author; year of publication; country; trial design; mean age; sample size; drug regimen during the experimental period; intervention characteristics, including the type of intervention, frequency, and duration; outcome measures and adverse events) of each article were extracted. The passive intervention was defined as the blank control in the control group, whereas the exact total training time in the experimental group was defined as the active intervention. Any conflicts or ambiguities in the reporting methods or results during data extraction were discussed with a third reviewer (WS) and resolved by consensus.

**Table 1:** Characteristics of randomized controlled trials included in the meta-analysis.

			**Participant characteristics**				**Intervention protocol**			
**References**	**Country**	**Study design**	**Age, mean (SD)**	**N (EG/CG)**	**H&Y**	**Med**	**Intervention**	**Control**	**Outcome**	**Adverse effects**
Schmitz-Hübsch et al. (2006)	Germany	RCT	EG:64(8) CG:63(8)	56 (32/24)	NA	ON	Qigong 1 × 90 min/week 16 weeks	No intervention	UPDRS-III	NR
Hackney and Earhart (2008)	USA	RCT	EG:64.9(8.3) CG:62.6(10.2)	23 (17/16)	1.5–3	ON	Tai Chi 2 × 60 min/week 13 weeks	No intervention	UPDRS-III; BBS; TUG; gait velocity; 6 MWT; stride length; OLBB	NR
Li et al. (2012)	USA	RCT	EG:68(9) CG1:69(8) CG2:69(9)	195 (65/65/65)	1–4	ON	Tai Chi 2 × 60 min/week 24 weeks	Stretching; resistance	UPDRS-III; TUG; gait velocity; stride length;	Fall (*n* = 11) Muscle pain (*n* = 6) Dizziness (*n* = 5) Hypotension (*n* = 4)
Amano et al. (2013)	USA	RCT	EG:66(11) CG:66(7)	24(15/9)	2–3	ON	Tai Chi 3 × 60 min/week 16 weeks	No intervention	UPDRS-III; gait velocity gait cadence; step length	NR
Choi et al. (2013)	Korea	RCT	EG:60.81(7.6) CG:65.54(6.8)	20 (11/9)	1–2	ON	Tai Chi 3 × 60 min/week 12 weeks	No intervention	UPDRS-III; TUG; 6 MWT; OLBB	NR
Gao et al. (2014)	China	RCT	EG:69.54(7.32) CG:68.28(8.53)	76 (37/39)	1–4	ON	Tai Chi 3 × 60 min/week 12 weeks	No intervention	UPDRS-III; BBS; TUG	NR
Zhang et al. (2015)	China	RCT	EG:66(11.8) CG:64.35(10.53)	40 (20/20)	1–3	ON	Tai Chi 2 × 60 min/week 12 weeks	Multimodal exercise training	UPDRS-III; BBS; TUG; gait velocity; stride length	NR
Xiao and Zhuang (2016)	China	RCT	EG:66.52(2.13) CG:68.17(2.27)	89 (45/44)	1–3	ON	Qigong (Baduanjin) 4 × 75 min/week 24 weeks	Walking	UPDRS-III; BBS; TUG; Gait velocity; 6 MWT; stride length	NR
Xiao et al. (2016)	China	RCT	EG/CG:67.8(9.4)	68(35/33)	NA	ON	Qigong (Baduanjin) 4 × 60 min/week 24 weeks	Conventional training	UPDRS-III; BBS; TUG; Gait velocity; 6 MWT	NR
Liu et al. (2016)	China	RCT	EG:65.84(5.45) CG:62.5(3.13)	41(23/18)	NA	ON	Qigong 5 × 60 min/week 10 weeks	Daily activities	One legged blind balance; TUG	NR
Lee et al. (2018)	Korea	RCT	EG:65.8(7.2) CG:65.7(6.4)	41(25/16)	1–3	ON	Qigong (QI Dance) 2 × 60 min/week 8 weeks	No intervention	UPDRS-III; BBS	NR
Kurt et al. (2018)	Turkey	RCT	EG:62.41 (6.76) CG:63.61(7.18)	40(20/20)	2–3	ON	Tai Chi (Ai Chi) 5 × 60 min/week 5 weeks	Land-based exercise	UPDRS-III; berg; TUG	NR
Vergara-Diaz et al. (2018)	USA	RCT	EG:65.7(3.86) CG:62.0(7.77)	25(12/13)	2–2.5	OFF	Tai Chi 2 × 60 min/week 24 weeks	Usual healthcare	UPDRS-III; TUG; gait velocity;	NR
Wan et al. (2021)	China	RCT	EG:64.95(7.83) CG:67.03(7.47)	40(20/20)	1–4	ON	Qigong 4 × 60 min/week 12 weeks	No intervention	TUG; gait velocity; stride length; gait cadence; OLBB	NR
Li et al. (2022)	China	RCT	EG:62.7(5.51) CG1:61.9(5.64) CG2:61.9(6.76)	95(32/31/32)	1–2.5	ON	Tai Chi 2 × 60 min/week 48 weeks	Brisk walking; no intervention	UPDRS-III; BBS; TUG; gait velocity; gait cadence; stride length; step length	NR

*UPDRS-III, Unified Parkinson's Disease Rating Scale part III; BBS, Berg Balance Scales; 6 MWT, 6 min walking test; TUG, time up and go test; OLBB, one legged blind balance; H&Y, Hoehn and Yahr stage; NA, not available; Med, anti-Parkinson medication (“OFF” refers to medication off during measurement); NR, no report; EG, experimental group; CG, control group*.

We assessed the methodological quality of each included trial using the Physiotherapy Evidence Database (PEDro) scale (Maher et al., 2003), which consists of 11 items to evaluate the quality of studies. It scores trials ranging from 0 (low quality) to 10 (high quality), yet, the first item (eligibility criterion) is excluded from the total score as it is required to prove external validity. A score of 6 is considered the cut-off for high-quality trials (Moher et al., 2009). The Cochrane Collaboration's tool (Higgins et al., 2011) was used to assess the risk of bias in each study. This tool covers sequence generation, allocation concealment, blinding of participants, personnel, and outcome assessors, incomplete outcome data, selective outcome reporting, and other sources of bias. We divided objects into three categories: “low risk,” “high risks,” and “unclear risk.” Two authors (MW and WS) completed the scoring process, and any differences that arose throughout the evaluation were reviewed by a third reviewer (LT) and resolved by consensus. Each study's scores were unanimously agreed upon and summarized in Table 2.

**Table 2:** Physiotherapy Evidence Database (PEDro) scores of the 15 included studies.

**References**	**Scores**	**Methodological quality**	**PEDro item number**										
			**1**	**2**	**3**	**4**	**5**	**6**	**7**	**8**	**9**	**10**	**11**
Schmitz-Hübsch et al. (2006)	8	Good	1	1	1	1	0	0	1	1	1	1	1
Hackney and Earhart (2008)	6	Good	1	1	0	1	0	0	1	0	0	1	1
Li et al. (2012)	8	Good	1	1	1	1	0	0	1	1	1	1	1
Amano et al. (2013)	6	Good	1	1	0	1	0	0	1	1	0	1	1
Choi et al. (2013)	6	Good	1	1	0	1	0	0	1	1	0	1	1
Gao et al. (2014)	7	Good	1	1	1	1	0	0	1	1	0	1	1
Zhang et al. (2015)	8	Good	1	1	1	1	0	0	1	1	1	1	1
Xiao and Zhuang (2016)	7	Good	1	1	0	1	0	0	1	1	1	1	1
Xiao et al. (2016)	5	Fair	1	1	0	1	0	0	0	1	0	1	1
Liu et al. (2016)	4	Fair	1	1	0	1	0	0	0	0	0	1	1
Lee et al. (2018)	7	Good	1	1	1	1	0	0	1	0	1	1	1
Kurt et al. (2018)	6	Good	1	1	1	1	0	0	0	1	0	1	1
Vergara-Diaz et al. (2018)	6	Good	1	1	1	1	0	0	0	1	0	1	1
Wan et al. (2021)	5	Fair	1	1	1	1	0	0	0	0	0	1	1
Li et al. (2022)	7	Good	1	1	1	1	0	0	1	0	1	1	1

*Studies were classified as having excellent (9–10), good (6–8), fair (4–5), or poor (< 4). 0: does not meet the criteria; 1: meets the criteria. Criteria (without eligibility criteria) were used to calculate the total PEDro score; Item 1 = Eligibility criteria; Item 2 = Random sequence; Item 3 = Allocation concealment; Item 4 = Similar at baseline; Item 5 = Subjects blinded; Item 6 = Therapists blinded; Item 7 = Assessors blinded; Item 8 = < 15% dropouts; Item 9 = Intention-to-treat analysis; Item 10 = Between-group comparisons; Item 11 = Point measures and variability data*.

### Statistical Analysis

All analyses were conducted using Comprehensive Meta-analysis Version 2.2 software (Biostat Inc., Englewood, NJ, USA). We used an inter-group, pre- to post-intervention, meta-analysis design based on standardized mean differences (Hedges' g). In the total estimated effect sizes (ESs), the random effects model was utilized with a 95% confidence interval (CI) to avoid the high likelihood of false-positive results (Heung-Sang Wong et al., 2017). Hedges' g, a variant of Cohen's d that corrects for sample size biases, was used to calculate the ESs. The ES was categorized as follows in accordance with the Cochrane's handbook: small (0.2–0.49), moderate (0.5–0.79), or large (0.8 or more) (Higgins et al., 2003). A positive ES indicated a more favorable outcome for the experimental group. The I^2^ statistic estimated heterogeneity among studies and classified as 25% (low heterogeneity), 50% (moderate heterogeneity), or 75% (high heterogeneity) (Higgins et al., 2003). If the data was unsuitable for our analysis, the previous statistical formula was used to convert the data into mean and SD format (Hozo et al., 2005). The funnel plot and Egger's regression test were used to identify publication bias. The impact of publication bias on the pooled results was further investigated using a 'trim and fill' strategy in the event of publication bias. A sensitivity analysis was also performed to detect the presence of highly influential studies that could skew the results. Studies were deemed influential if their removal significantly modified the summary effect (i.e., from significant to non-significant). The significance level was set at *p* ≤ 0.05.

Finally, all moderators (methodological quality, type of control group, exercise type, exercise duration, exercise frequency, and number of sessions) were implemented as categorical variables. The meta-regressors were continuous variables related to TCE dosage [exercise frequency (sessions per week), exercise duration (weeks), number of sessions (n), and session duration (min)].

## Results

### Search Results

**Figure 1:**
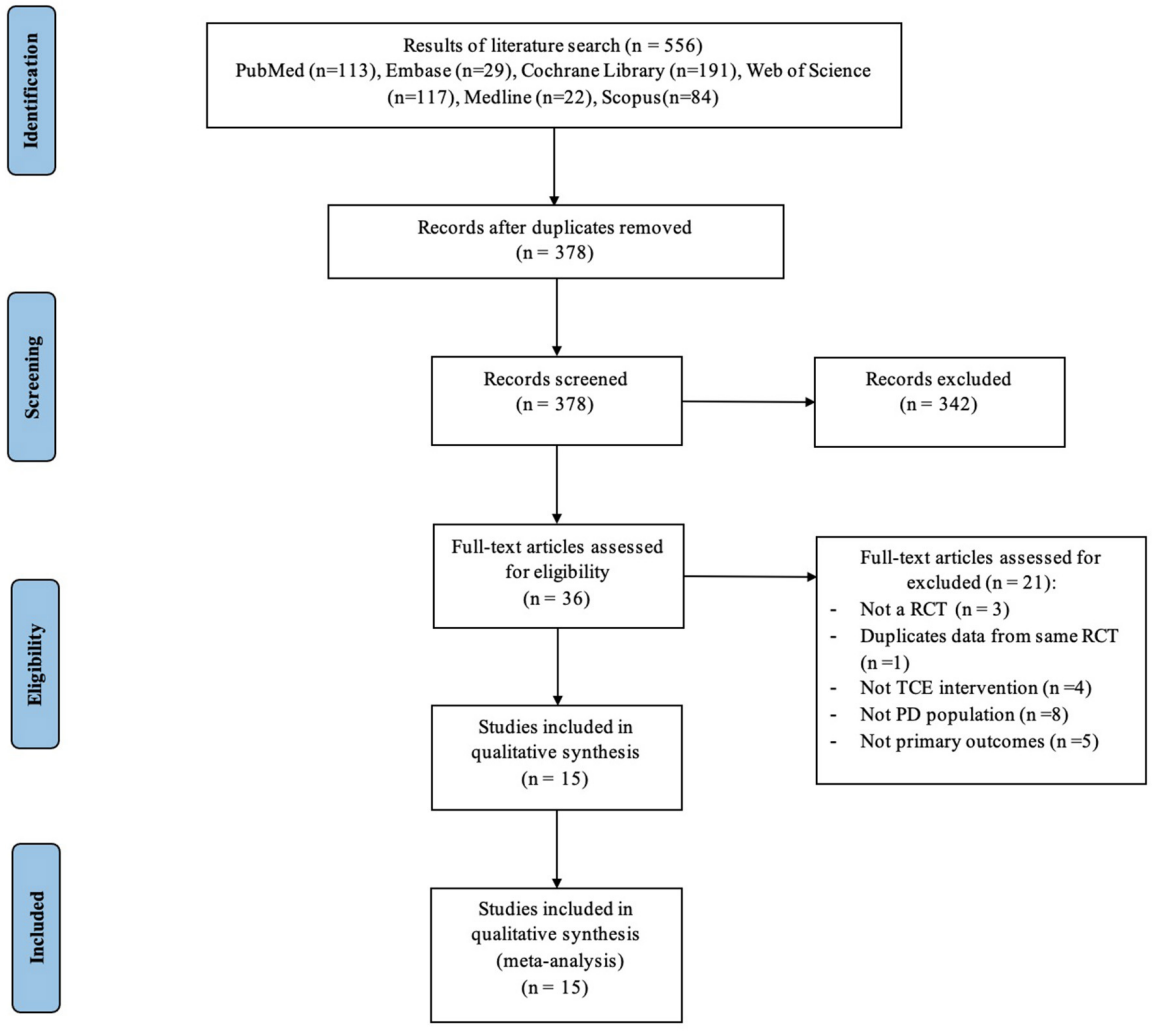
Process of study selection following the Preferred Reporting Items for Systematic Reviews and Meta-Analyses (PRISMA).

The flowchart in Figure 1 summarizes the PRISMA-compliant literature search and selection process. Our comprehensive review of the literature uncovered 556 studies, of which 378 remained after duplicates were deleted. We then examined the names and abstracts of the remaining papers, of which 342 were excluded. The remaining 36 studies were read in their entirety. Among these 36 articles, three were excluded because they were not RCTs, one presented duplicated data from a previous RCT, eight were excluded because participants did not meet the inclusion criteria, four did not use TCE intervention, and five did not have data access. Eventually, 15 RCTs (Schmitz-Hübsch et al., 2006; Hackney and Earhart, 2008; Li et al., 2012, 2022; Amano et al., 2013; Choi et al., 2013; Gao et al., 2014; Zhang et al., 2015; Liu et al., 2016; Xiao and Zhuang, 2016; Xiao et al., 2016; Kurt et al., 2018; Lee et al., 2018; Vergara-Diaz et al., 2018; Wan et al., 2021) were deemed eligible for inclusion in the meta-analysis. The two researchers have the same rate of research selection and data extraction as 4 and 82%, respectively.

### Study Characteristics

Table 1 lists the characteristics of each of the included studies, which were published between 2006 and 2022. Seven studies were conducted in China (Gao et al., 2014; Zhang et al., 2015; Liu et al., 2016; Xiao and Zhuang, 2016; Xiao et al., 2016; Wan et al., 2021; Li et al., 2022), four in the USA (Hackney and Earhart, 2008; Li et al., 2012; Amano et al., 2013; Vergara-Diaz et al., 2018), two in Korea (Choi et al., 2013; Lee et al., 2018), one in Germany (Schmitz-Hübsch et al., 2006), and one in Turkey (Kurt et al., 2018). The TCE program was used to treat PD in all experimental groups, with nine studies using Tai Chi and six studies using Qigong. Active (e.g., brisk walking, stretching, resistance, or usual healthcare) or passive interventions (i.e., no intervention, wait-list) were employed in the control group. These participants were prescribed 75–90 min of exercise in each session one to five times per week for 5–48 weeks. The outcomes of these 15 studies were as follows: Unified Parkinson's Disease Rating Scale part III (UPDRS-III), Berg Balance Scales (BBS), time up and go (TUG), one-legged blind balance test, gait velocity, 6-min walking test (6 MWT), stride length, cadence, and step length. One study (Li et al., 2012) reported on suspected side effects such as dizziness (*n* = 5), muscle pain (*n* = 6), and symptoms of hypotension (*n* = 4). The remaining studies reported no TCE-related side effects.

### Study Quality

**Figure 2:**
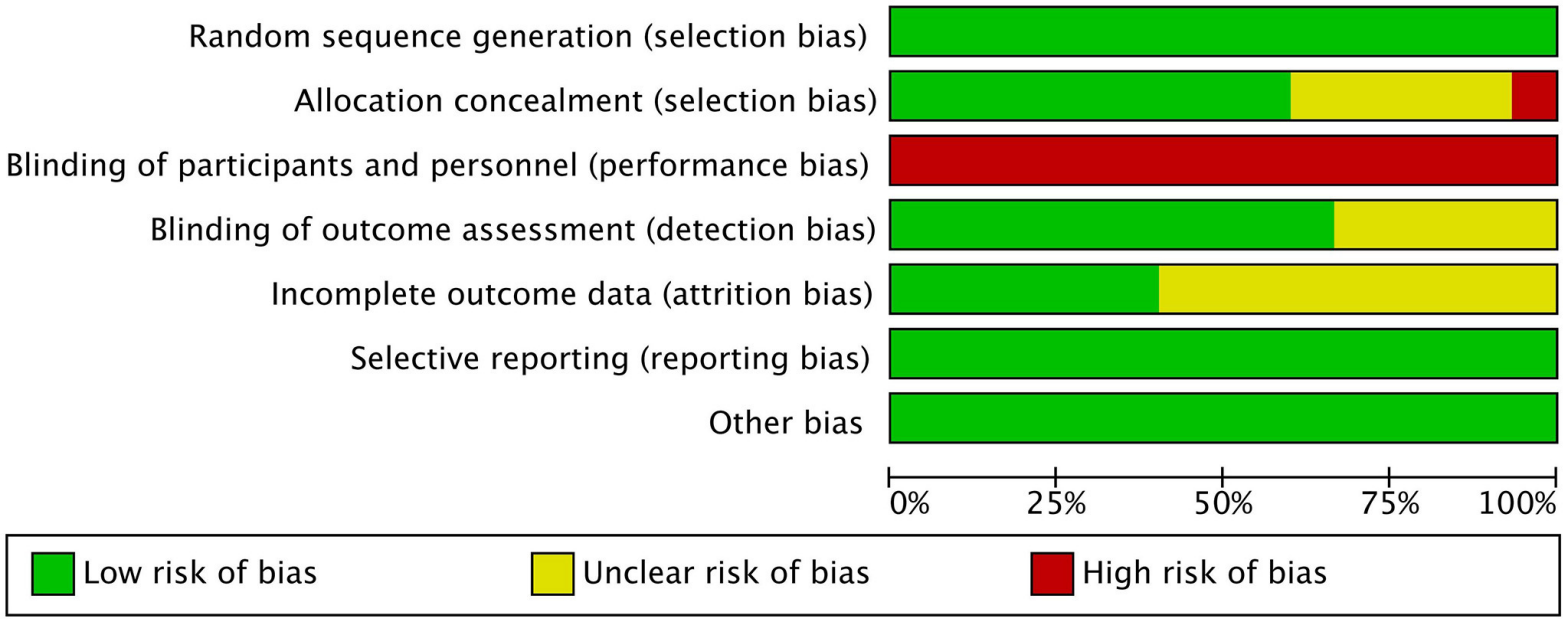
Assessment of risk of bias with selected studies.

Table 2 presents the methodology quality of the included studies. The quality of the studies ranged between fair and good (score range: 5–9 points), with 80% of studies being classified as good quality and 12% as fair quality. Nine studies used a concealed allocation procedure (Schmitz-Hübsch et al., 2006; Li et al., 2012, 2022; Gao et al., 2014; Zhang et al., 2015; Kurt et al., 2018; Lee et al., 2018; Vergara-Diaz et al., 2018; Wan et al., 2021), and all reported random assignment. We could not blind patients and therapists because this was an interventional movement study. However, 10 trials blinded the outcome assessors (Schmitz-Hübsch et al., 2006; Hackney and Earhart, 2008; Li et al., 2012, 2022; Amano et al., 2013; Choi et al., 2013; Gao et al., 2014; Zhang et al., 2015; Xiao and Zhuang, 2016; Lee et al., 2018). Five studies had a dropout rate of >85% (Hackney and Earhart, 2008; Liu et al., 2016; Lee et al., 2018; Wan et al., 2021; Li et al., 2022). Nine studies in particular did not analyze missing data using intent-to-treat analyses (Hackney and Earhart, 2008; Amano et al., 2013; Choi et al., 2013; Gao et al., 2014; Liu et al., 2016; Xiao et al., 2016; Kurt et al., 2018; Vergara-Diaz et al., 2018; Wan et al., 2021). The risk of bias assessment of all included studies is shown in Figure 2.

### Synthetic Results

**Figure 3:**
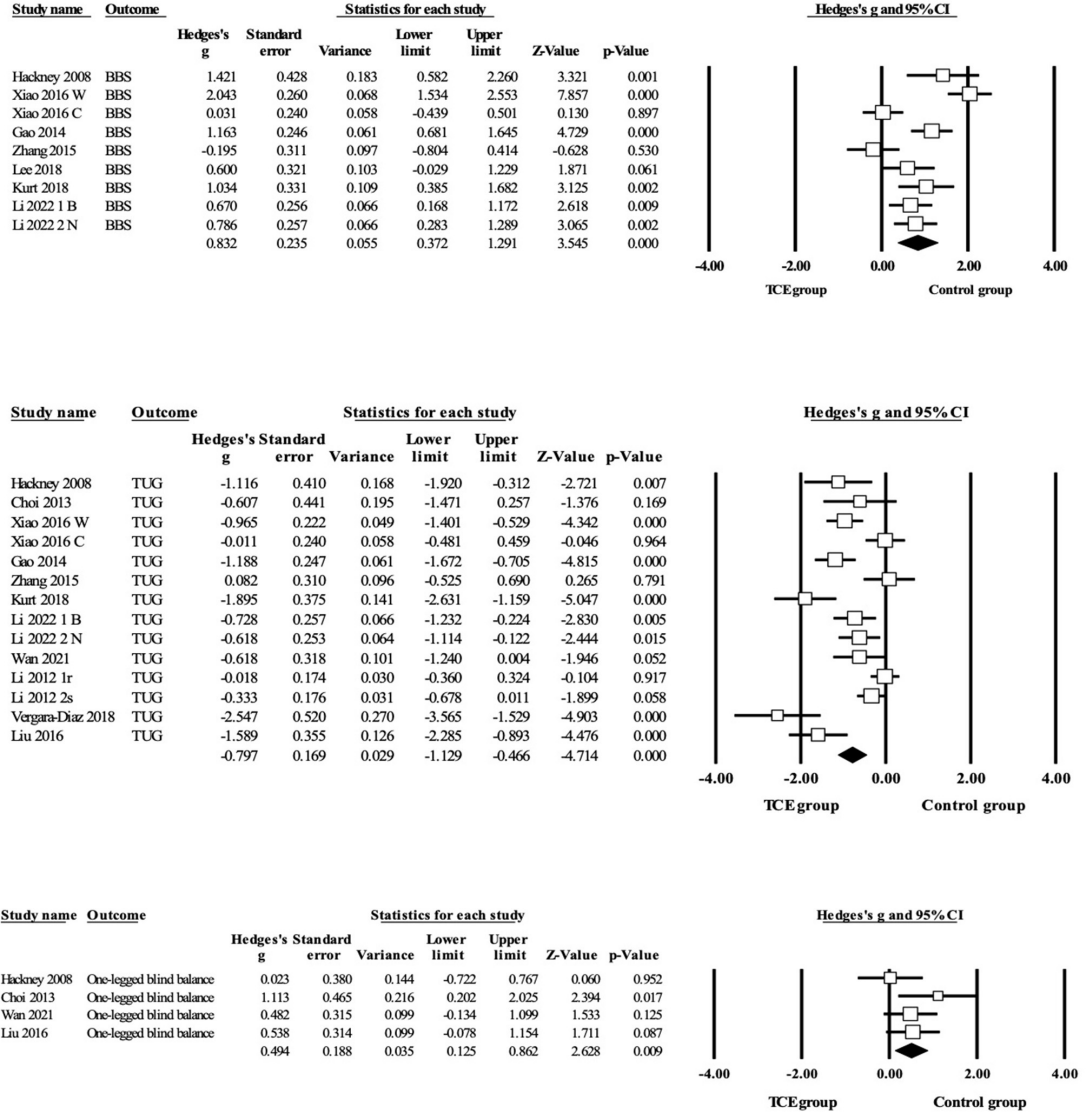
Forest plot showing the effects of TCE vs. control group on BBS outcomes: BBS, TUG, one-legged blind balance.

Regarding balance outcomes (Figure 3 and Table 3), the pooled data demonstrated that TCE resulted produced large and significant improvements in BBS when compared to the control group (*g* = 0.83, 95% CI = 0.37–1.29, *p* = 0.000, I^2^ = 84%) and TUG (*g* = −0.80, 95% CI = −1.13– −0.47, *p* = 0.000, I^2^ = 81%). Moreover, pooled analyses from four parallel trials revealed that the one-legged blind balance test exerted a small and significant increase in effect size (*g* = 0.49, 95% CI = 0.13–0.86, *p* = 0.01, I^2^ = 10%) compared with the control group.

**Table 3:** Synthesized results for the effects of TCE vs. control group intervention.

**Variables**	**k**	**g**	**95% CI**	**I^**2**^ %**	**Between-group homogeneity**			**Publication bias Egger's test (*p*)**
					**Q-value**	**df (Q)**	***p*** **-value**	
**Balance outcomes**								
BBS	9	0.83	0.64–1.00	84	48.78	8	0.000	0.43
TUG	14	−0.80	−1.13– −0.47	81	68.22	13	0.000	0.01
OLBB	4	0.49	0.13–0386	10	3.33	3	0.34	0.30
**Gait outcomes**								
Gait velocity	11	0.28	0.02–0.54	64	27.77	10	0.002	0.46
6 MWT	4	0.32	0.01–0.62	15	3.55	3	0.32	0.35
Stride length	8	0.25	0.08–0.41	42	12.09	7	0.10	0.17
Cadence	4	−0.03	−0.31–0.25	0	3.02	3	0.82	0.37
Step length	3	0.02	−0.41–0.45	41	3.39	2	0.18	0.50
**Motor symptoms**								
UPDRS-III	15	−0.77	−1.06– −048	76	58.39	14	0.000	0.45

*UPDRS-III, Unified Parkinson's Disease Rating Scale part III; BBS, Berg Balance Scales; OLBB, one legged blind balance; 6 MWT, 6 min walking test; TUG, time up and go test*.

**Figure 4:**
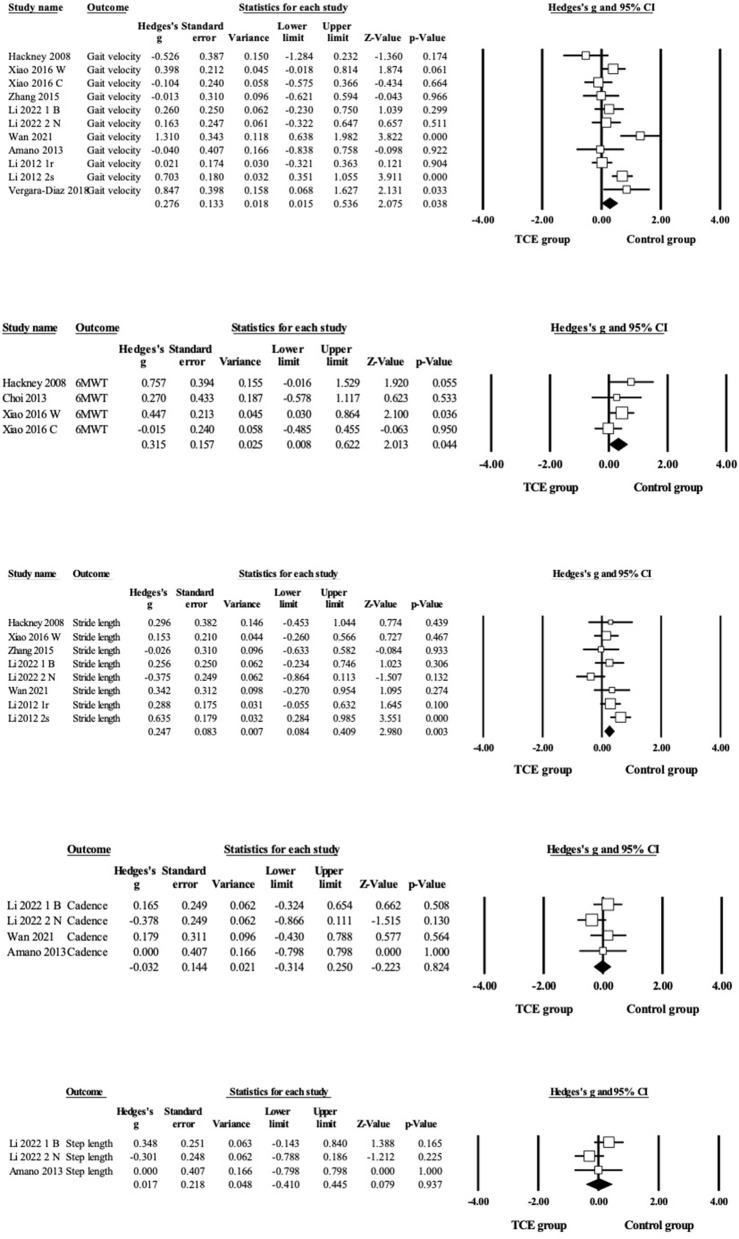
Forest plot showing the effects of TCE vs. control group on gait outcomes: gait velocity, 6 MWT, stride length, cadence, step length.

Regarding gait outcomes (Figure 4 and Table 3), the pooled results showed that TCE caused small and significant improvements in gait velocity (*g* = 0.28, 95% CI = 0.02–0.54, *p* = 0.04, I^2^ = 64%), 6MWT (*g* = 0.32, 95% CI 0.01–0.62, *p* = 0.04, *I*^2^= 15%) and stride length (*g* = 0.25, 95% CI = 0.08–0.41, *p* = 0.003, I^2^ = 42%) compared with the control group. However, no significant results were found for TCE intervention on cadence (*g* = −0.03, 95%CI = −0.31–0.25, *p* = 0.82, I^2^ = 0%) and step length (*g* = 0.02, 95%CI = −0.30–0.34, *p* = 0.92, I^2^ = 41%) compared with the control group.

**Figure 5:**
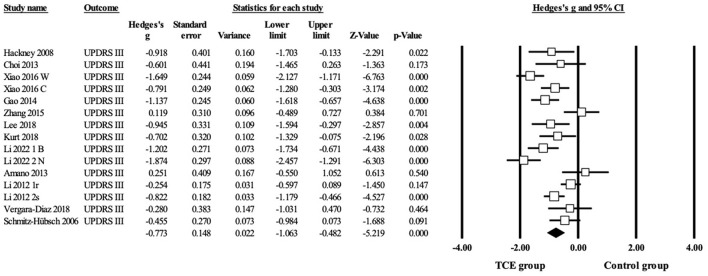
Forest plot showing the effects of TCE vs. control group on motor symptoms: UPDRS-III.

Regarding motor symptoms (Figure 5 and Table 3), 13 studies reported UPDRS-III scores, including 15 parallel comparisons between the TCE and control groups (as two studies included two paired trials each). Data from fifteen trials demonstrated a significant improvement in UPDRS-III compared with the control group (*g* = −0.77, 95% CI = −1.06– −0.48, *p* = 0.000, I^2^ = 76%).

According to the sensitivity analysis, no study significantly impacted the outcomes (data not shown). No study was deemed insignificant since its removal had no discernible effect on the overall effect (i.e., a change from significant to non-significant). Although there was considerable heterogeneity between RCTs, the moderators that increased the effect of TCE on balance, gait, and motor signs also reduced the analyses' heterogeneity.

### Moderator Analysis

The categorical and continuous variables in Table 4 were used to conduct moderator analyses. PEDro score, type of exercise, type of control group, and number of sessions significantly moderated the effects of TCE on BBS (Q = 48.79, *df* = 8, *p* = 0.000) and gait velocity (Q = 27.77, *df* = 10, *p* = 0.001). Compared to RCTs on TCE with low methodological quality (*g* = 0.68, 95% CI = −0.68–2.04, *p* = 0.33), RCTs on TCE with high methodological quality (*g* = 0.88, 95% CI = 0.38–1.38, *p* = 0.001) significantly improved BBS. Moreover, high-quality methodological RCTs (*g* = 0.31, 95% CI = 0.14–0.47, *p* = 0.000) of TCE significantly improved gait velocity compared with low-quality methodological RCTs on TCE (*g*= 0.23, 95% CI = −0.78–1.24, *p* = 0.66). Additionally, Tai chi (*g* = 0.79, 95% CI = 0.38–1.21; *p* = 0.001) significantly improved BBS compared with Qi gong (*g*= 0.89, 95% CI = −0.36–2.15; *p* = 0.16). The change in gait velocity due to Tai Chi (*g* = 0.28, 95% CI = 0.01–0.54, *p*= 0.04) was more significant than due to Qigong (*g* = 0.27, 95% CI = −0.37–0.92, *p* = 0.41). Active control groups (*g* = 0.27, 95% CI = 0.11–0.43, *p* = 0.001) exhibited significantly improved gait velocity compared with non-active control groups (*g* = 0.26, 95% CI = −0.87–1.39, *p* = 0.65). The number of sessions shows that the effect size of more than 48 sessions (*g*= 0.37, 95% CI= 0.09–0.64, *p* = 0.01) was moderate and significant compared with <48 sessions (*g* = −0.02.,95% CI = −0.21–0.28, *p* = 0.38).

**Table 4:** Moderator analysis for the effects of TCE on measurement outcomes.

**Variables**	**Balance outcomes**			**Gait outcomes**					**Motor symptoms**
	**BBS**	**OLBB**	**TUG**	**Gait velocity**	**6 MWT**	**Stride length**	**Cadence**	**Step length**	**UPDRS–III**
	**Hedges'*g* (95%CI)**	**Hedges'*g* (95%CI)**	**Hedges'*g* (95%CI)**	**Hedges'*g* (95%CI)**	**Hedges'*g* (95%CI)**	**Hedges'*g* (95%CI)**	**Hedges'*g* (95%CI)**	**Hedges'*g* (95%CI)**	**Hedges'*g* (95%CI)**
**PEDro score**	
≥6 <6	0.88 (0.38–1.38) 0.68 (−0.68 to 2.04)	1.11 (0.20–2.03) 0.39 (0.01–0.76)	−0.80 (−1.2– −0.41) −0.80 (−1.52– −0.08)	0.31 (0.14–0.47) 0.23 (−0.78–1.24)	0.41 (0.04–0.79) 0.31 (−0.44 to 1.05)	0.19 (−0.09–0.46) 0.32 (−0.15–0.80)	−0.09 (−0.41–0.23) 0.18 (−0.43–0.79)	0.02 (−0.30–0.34) –	−0.76 (−1.09– −0.43) −0.83 (−1.24– 0.41)
**Exercise type**									
Tai Chi Qigong	0.79 (0.38–1.21) 0.89 (−0.36 −2.15)	– 0.49 (0.14–0.84)	−0.82 (−1.23– −0.40) −0.77 (−1.40– −0.14)	0.28 (0.01–0.54) 0.27 (−0.37–0.92)	0.27 (−0.58–1.12) 0.34 (−0.07–0.74)	0.19 (−0.15–0.53) 0.23 (−0.09–0.54)	−0.09 (−0.41–0.23) 0.18 (−0.43–0.79)	0.02 (−0.30–0.34) –	−0.70 (−1.05– −0.35) −0.97 (−1.50– −0.44)
**Type of control group**									
Active control Non-active control	0.73 (0.09–1.37) 1.04 (0.69–1.38)	0.54 (−0.08–1.15) 0.49 (−0.06–1.04)	−0.77 (−1.19– −0.36) −0.95 (−1.27– −0.63)	0.27 (0.11–0.43) 0.26 (−0.87–1.39)	0.23 (−0.22–0.68) 0.54 (−0.04–1.11)	0.19 (−0.09–0.46) 0.32 (−0.15–0.80)	−0.11 (−0.45–0.24) 0.11 (−0.37–0.60)	0.02 (−0.33–0.37) –	−0.84 (−1.24– −0.43) −0.68 (−1.6– −0.29)
**Exercise frequency**									
≥3 sessions/week <3 sessions/week	1.07 (0.20–1.93) 0.62 (0.18–1.06)	−0.62 (0.23–1.02) 0.02 (−0.72–0.77)	−0.96 (−1.43– −0.49) −0.62 (−1.06– −0.19)	0.38 (−0.19–0.60) 0.23 (−0.08–0.53)	0.76 (−0.02–1.53) 0.25 (−0.06–0.55)	0.21 (−0.13–0.55) 0.21 (−0.09–0.50)	0.11 (−0.37–0.60) −0.11 (−0.64–0.43)	– 0.02 (−0.61–0.66)	−0.83 (−1.31– −0.36) −0.73 (−1.11– −0.36)
**Exercise duration**									
>12weeks ≤ 12weeks	0.97 (0.26–1.69) 0.66 (0.05–1.27)	0.02 (−0.72–0.77) 0.62 (0.23–1.02)	−0.67 (−1.06– −0.28) −0.96 (−1.54– −0.39)	0.22 (−0.03–0.46) 0.64 (−0.66–1.94)	0.32 (0.03–0.61) 0.27 (−0.58–1.12)	0.23 (−0.05–0.50) 0.16 (−0.28–0.59)	−0.09 (−0.41–0.23) 0.18 (−0.43–0.79)	0.02 (−0.41–0.45) –	−0.82 (−1.20– −0.44) −0.67 (−1.13– −0.21)
**Number of sessions**									
<48 sessions ≥48 sessions	0.79 (0.24–1.34) 0.88 (0.05–1.71)	0.50 (0.05–0.92) 0.48 (−0.13–1.10)	−1.05 (−1.63– −0.47) −0.61 (−0.98– −0.24)	−0.21 (−0.71–0.28) 0.37 (0.09–0.64)	0.54 (−0.04–1.11) 0.23 (−0.22–0.68)	0.10 (−0.37–0.57) 0.24 (−0.03–0.50)	– −0.03 (−0.31–0.25)	– 0.02 (−0.41–0.45)	−0.68 (−0.91– −0.45) −0.85 (−1.30– −0.40)

*UPDRS-III, Unified Parkinson's Disease Rating Scale part III; BBS, Berg Balance Scales; OLBB, one legged blind balance; 6MWT, 6min walking test; TUG, time up and go test*.

### Meta-regression

In the meta-regression (Table 5 and Supplementary Material), covariates that significantly affected UPDRS-III included exercise duration (β = −0.0224, 95% CI = −0.0443– −0.0006; *p* = 0.044) and number of sessions (β = −0.0108, 95% CI = −0.0190– −0.0026; *p* = 0.01) in random-effect regression analyses. Session duration was a significant covariate (β = 0.0920, 95% CI = 0.0212– – 0.1628; *p* = 0.011) on BBS in regression analyses. However, we found that exercise frequency (sessions per work), exercise duration, number of sessions and session duration did not impact other outcomes significantly (*P* > 0.05).

**Table 5:** Meta-regression of the 15 included studies to predict TCE effects on measurement outcomes.

**Variables**	**Balance outcomes**			**Gait outcomes**				**Motor symptoms**
	**BBS**	**OLBB**	**TUG**	**Gait velocity**	**6 MWT**	**Stride length**	**Cadence**	**UPDRS–III**
	**β (95% CI)**	**β (95% CI)**	**β (95% CI)**	**β (95% CI)**	**β (95% CI)**	**β (95% CI)**	**β (95% CI)**	**β (95% CI)**
Exercise frequency (sessions per week)	0.1629 (−0.2710–0.5968)	0.0815 (−0.2583–0.4213)	−0.2382 (−0.5179–0.0415)	−0.1059 (−0.4408–0.2291)	−0.2282 (−0.6442–0.1878)	−0.0359 (−0.3755–0.3036) 0.84	0.0291 (−0.9268–0.9850)	−0.0973 (−0.3788–0.1842)
Exercise duration (weeks)	−0.0013 (−0.0332–0.0306)	−0.1031 (−0.4830–0.2768)	0.0125 (−0.0136–0.0386)	−0.0014 (−0.0239–0.0211)	−0.0248 (−0.0895–0.0400)	−0.0094 (−0.0260–0.0072)	−0.0097 (−0.0255–0.0125)	−0.0224 (−0.0443– −0.0006)
Number of sessions (*n*)	0.0019 (−0.0120–0.0157)	0.0126 (−0.0306–0.0558)	0.0052 (−0.0073–0.0177)	−0.000 (−0.0100–0.0099)	−0.0049 (−0.0159–0.0061)	−0.0045 (−0.0121–0.0032)	−0.0045 (−0.0183–0.0092)	−0.0108 (−0.0190– −0.0026)
Session duration (min)	0.0920 (0.0212–0.1628)	–	−0.0119 (−0.0961–0.0724)	0.0091 (−0.0505–0.0688)	0.0128 (−0.0378–0.0634)	−0.0048 (−0.0501–0.0405)	–	−0.0043 (−0.0403–0.0318)

*UPDRS-III, Unified Parkinson's Disease Rating Scale part III; BBS, Berg Balance Scales; OLBB, one legged blind balance; 6 MWT, 6 min walking test; TUG, time up and go test*.

### Publication Bias

The Egger's test (Table 3) and funnel plot (Supplementary Material) were used to assess publication bias. The asymmetrical distribution of the included trials on a funnel plot revealed publication bias for RCTs that reported TUG. The Egger's test showed that Egger's regression intercept = −4.68, *p* < 0.05, and the Duval and Tweedie's trim and fill revealed that five studies were missing on the right side of the mean effect. The adjusted value was as follows: g= −0.38 (95% CI = −0.50– −0.25). As studies with smaller effect sizes were excluded from this meta-analysis, there may be evidence of publication bias.

## Discussion

The current systematic review with meta-analysis showed that TCE significantly improved balance outcomes (BBS, TUG, and one-legged blind balance), gait outcomes (gait velocity, 6 WMT, and stride length), and motor symptoms (UPDRS-III) compared with the control group in individuals with PD. Data from 15 RCTs involving a total of 873 participants were analyzed. The moderator analyses showed the effects of TCE on BBS and gait velocity were moderated by the PEDro score, exercise type, control group type, and number of sessions. Lastly, meta-regression revealed significant relationships between TCE dosage variables (exercise duration, number of sessions, session duration) and observed changes in UPDRS-III and BBS. The safety of the intervention should also be evaluated in future RCTs, regardless of the limited number of reported adverse events.

Common testing tools for balance outcomes include BBS, TUG, and the one-legged blind balance test. Clinicians frequently utilize the TUG test to assess the balance and mobility of patients with movement disorders and the elderly. The time necessary to maintain the body's center of gravity on a single support surface without visual cues is reflected in the one-legged blind balance test. People with PD are more likely to fall due to their movement abnormalities (Lomas-Vega et al., 2017). Strengthening the static and dynamic balancing abilities of patients prone to falls can improve muscle strength in their lower limbs and prevent falls. Tai Chi incorporates a variety of exercises, such as slow weight shifting, body rotation, and single-leg standing in various postures, all of which necessitate sensitive joint control and muscle coordination, resulting in increased proprioceptive stimulation and lower extremity muscle strengthening. Flexion and extension, raising and lowering, opening and shutting the trunk of the limbs, altering breathing according to the action, keeping balance, and continually changing the center of gravity are all techniques used in fitness Qi gong (Subramanian, 2017; Penn et al., 2019). Also, given that a previous review assessed the effect of single TCE on walking ability and balance (Yang et al., 2015; Winser et al., 2018; Wang et al., 2021), the current study found similar results, indicating that TCE could help patients with PD to improve their balance. It reduces nerve exhaustion, relaxes the neural system, strengthens core muscles, enhances joint activity, and improves proprioceptive input to the trunk and lower limbs, all of which help individuals with PD to improve their balance. These findings also suggest that single TCE can help people to increase their aerobic endurance and reduce their chance of falling by improving their balance (Winser et al., 2018). However, they did not apply meta-regression analysis to analyze the effect of factors on trial effect size. Even though they had small sample sizes, none of them calculated effect sizes using Hedges' g statistic. Thus, our findings pool the effects of multiple common TCEs in balance outcomes and reinforce the important role of balance outcomes in individuals with PD.

Concerning the gait outcomes, kinematic gait metrics (such as gait velocity, stride length, and 6 MWT) improved significantly, whereas gait cadence and step length remained the same. The routines chosen from traditional Chinese training can also improve leg muscle strength effectively by focusing on frequent lower limb movements. The research revealed that the gastrocnemius and tibialis anterior muscles substantially impact gait in individuals with PD (Plotnik et al., 2005). These workouts help to improve gait stability, stride length, and gait speed. According to our meta-analysis, the gait velocity and stride length improved following TCE in those with PD, contradicting the results of earlier systematic reviews (Ni et al., 2014; Yang et al., 2014). In contrast, our findings provide more compelling evidence for various reasons. First, all included research had a moderate methodological quality according to the PEDro scale tool. In contrast, most of Ni et al.'s evaluation studies were of low methodological quality (Ni et al., 2014). Second, by including just RCTs in our meta-analysis, we could estimate the magnitude of the effect. Although some studies assessed by Yang et al. were of moderate quality (Yang et al., 2014), the small number of studies and the type of single intervention. Moreover, one of the eight studies in Yang et al.'s meta-analysis had a non-RCT study. Therefore, more research into the benefits of TCE interventions on gait function in people with PD is warranted.

The UPDRS-III, which tracks PD motor performance and disability level, is an important outcome measure for evaluating long-term training benefits for motor symptoms. The findings of this meta-analysis revealed that the motor symptoms of individuals with PD improved significantly after TCE. More precisely, TCE moderately affected UPDRS-III (g = −0.77). The magnitude of effects of TCE on UPDRS-III is similar to that reported previously (Song et al., 2017; Tang et al., 2019). However, their reviews did not analyze the potential moderators. We discovered moderators that influenced motor symptom correspondence in our studies of motor signs with moderator analysis; however, we need to confirm this conclusion with a larger sample size in the future. Given that UPDRS-III scores are expected to deteriorate with time in patients with PD, the improvements in UPDRS-III scores suggest the possibility of disease-modifying effects (Chung et al., 2020). Furthermore, muscle tension caused by uncoordinated contractions of active and antagonistic muscles affects a range of motor symptoms in patients with PD. During TCE, constant motor alterations and stimulation can increase muscle activity, resulting in improved motor performance (Hawley et al., 2014; Luo et al., 2017).

Our moderator analysis revealed that the effect of TCE on BBS was significantly higher (g = 0.88) when only high-quality methodological RCTs were analyzed, but not when low-quality methodological RCTs were analyzed (g = 0.58). Additionally, high-quality methodological RCTs (g = 0.31) have a greater effect on gait velocity than low-quality methodological RCTs (g = 0.23). In clinical research, high-quality RCTs are the primary source of evidence for the safety and efficacy of clinical therapies. They aid in avoiding or mitigating the risk of bias in these trials (Vinkers et al., 2021). As a result, our findings imply that the significant effects of TCE on BBS and gait speed were not based on low-quality RCTs; therefore future studies must utilize high-quality RCTs to evaluate the effects of TCE on BBS and gait speed in PD. As for the type of exercise, BBS and gait movements improved significantly in patients with PD after they performed Tai Chi exercises compared to Qigong exercises. Moderator analysis showed that TCE increased the effect on gait velocity compared with active control groups (g = 0.27). Non-active control groups may be ethically untenable as individuals with PD undergo progressive functional decline over time (Kwakkel et al., 2007). Thus, we believe that active control groups should be used as a comparator in future RCTs. Moderator analysis revealed that long sessions affected (g = 0.37) gait velocity more significantly than short sessions (g = −0.21). This repetitive exercise in motivating surroundings enables patients to become accustomed to comparable tasks over time, allowing them to regain their gait function.

Furthermore, our meta-regression demonstrated that exercise duration and the number of sessions changed in UPDRS-III, meaning that high-intensity long sessions improved UPDRS-III more than low-intensity short sessions after the exercise. A high dose of TCE enhances caudate dopamine release and neurotrophic factor expression, improving the functional connectivity of brain motor circuits and motor skill learning in people with PD (Sacheli et al., 2019). These neurophysiological improvements may have a long-term effect on brain function, reducing motor symptoms and balance function. In terms of session duration, intense training can provide enough of a training effect on BBS to patients with PD. This finding may be equivocal because most of these studies used extended session durations. Further studies are warranted to investigate session duration on the effects of TCE on balance function.

Our systematic review and meta-analysis have some advantages that should be mentioned. It followed the PRISMA statement to the letter, and our review methodology was registered. Only the RCT design was chosen due to its reliability. When the included trials had small sample sizes, we used Hedges' g to ensure an accurate estimation of the overall effect size. Other potential confounding variables were explored to determine their impact on the effects of TCE. Moreover, we decided to investigate the impact of covariates on the size of trial effects using a random-effects meta-regression model. This novel circumstance may provide additional data for future studies examining the influence of these confounding variables. These strengths contribute to the comprehensiveness and generalizability of our findings.

The current systematic review, however, has several limitations. First, the sample sizes and number of studies evaluating the effects of TCE on executive function and execution subcomponents were both insufficient to assess the effects. Second, we did not examine the long-term effects of TCE treatment on gait and balance outcomes with follow-up data. Third, most studies did not employ a blinding method (i.e., assessor blinding), resulting in subjective expectation bias; however, this is not a limitation of our meta-analysis but rather a problem of studies undertaken on this issue in general. Thus, performance bias was likely inescapable. Furthermore, large sample studies are required and long-term effects should be investigated to gain insight into prospective maintenance effects. An effective exercise program should be established as a promoting strategy in the treatment of PD.

## Conclusion

Our systematic review found that TCE improved motor symptoms, balance function, and gait function (e.g., gait velocity, 6 MWT, and stride length) in people with PD. Moreover, it did not affect gait cadence and step length. More extensive trials and more rigorous study designs are needed to strengthen the evidence. Lastly, future studies will be able to assess the long-term impact of TCE-based rehabilitation training to ensure its long-term sustainability.

## Data Availability Statement

The original contributions presented in the study are included in the article/Supplementary Material, further inquiries can be directed to the corresponding author.

## Author Contributions

MW, QT, LW, and LZ conceptualized the study. MW, MZ, WS, and LT selected, extracted, and analyzed data. LZ, QT, and LW supervised the study. MW, MZ, and WS drafted the manuscript. MW, LZ, and QT participated in the whole process. LZ and QT made final decisions. All authors contributed to the manuscript's writing and read and approved the final version.

## Funding

This work was supported by grants from the Natural Science Foundation of Heilongjiang Province (JJ2020LH1369); the Applied Technology Research and Development Program of Heilongjiang Province (GA19C110); the National Key Research and Development Program (2019YFC1710304).

## Conflict of Interest

The authors declare that the research was conducted in the absence of any commercial or financial relationships that could be construed as a potential conflict of interest.

## Publisher's Note

All claims expressed in this article are solely those of the authors and do not necessarily represent those of their affiliated organizations, or those of the publisher, the editors and the reviewers. Any product that may be evaluated in this article, or claim that may be made by its manufacturer, is not guaranteed or endorsed by the publisher.

## Abbreviations

PD: Parkinson's disease
TCE: traditional Chinese exercise
RCTs: randomized controlled trials
PEDro: Physiotherapy Evidence Database
CI: confidence interval
ES: effect size
UPDRS-III: Unified Parkinson's Disease Rating Scale part III
BBS: Berg Balance Scales
TUG: time up and go
6MWT: 6-minute walking test.
