# Supplementary material for: Efficacy of Traditional Chinese Exercise in Improving Gait and Balance in Cases of Parkinson's Disease: A Systematic Review and Meta-analysis
Source: Front Aging Neurosci. 2022 Jun 30;14:927315. doi: 10.3389/fnagi.2022.927315 (PMC9285003; doi:10.3389/fnagi.2022.927315)
Supplement: Supplementary file 1 [file Data_Sheet_1.DOCX]

Supplementary Material

**1. Supplementary Figures**

**Supplementary Appendix – Electronic searches**

**Pubmed**

("Tai-ji" [MeSH Terms] OR "Tai-ji" [Title/Abstract] OR "tai chi" [Title/Abstract] OR "chi tai" [Title/Abstract] OR "tai ji quan" [Title/Abstract] OR "ji quan tai" [Title/Abstract] OR "quan tai ji" [Title/Abstract] OR "Taiji" [Title/Abstract] OR "Taijiquan" [Title/Abstract] OR "t'ai chi" [Title/Abstract] OR "tai chi chuan" [Title/Abstract] OR ("qigong" [MeSH Terms] OR "qi gong" [Title/Abstract] OR "ch'i kung" [Title/Abstract] OR "Yijinjing" [Title/Abstract] OR "Baduanjin" [Title/Abstract] OR "Wuqinxi" [Title/Abstract]) OR "traditional Chinese exercise" [Title/Abstract]) AND ("Parkinson Disease" [Mesh Terms] OR "Idiopathic Parkinson's Disease" [Title/Abstract] OR "Lewy Body Parkinson's Disease" [Title/Abstract] OR "Parkinson's Disease Idiopathic" [Title/Abstract] OR "Parkinson's Disease Lewy Body" [Title/Abstract] OR "Parkinson Disease Idiopathic" [Title/Abstract] OR "Parkinson's Disease" [Title/Abstract] OR "Idiopathic Parkinson Disease" [Title/Abstract] OR "Lewy Body Parkinson Disease" [Title/Abstract] OR "Primary Parkinsonism" [Title/Abstract] OR "Parkinsonism Primary" [Title/Abstract] OR "Paralysis Agitans" [Title/Abstract]) AND ("randomized controlled trials" [Title/Abstract] OR "clinical trial" [Title/Abstract] OR "randomization" [Title/Abstract] OR "randomized" [Title/Abstract])

**Scopus**

#1 TITLE-ABS-KEY ("Tai-ji" OR "Tai Chi" OR "Chi, Tai" OR "Tai Ji Quan" OR "Ji Quan, Tai" OR "Quan, Tai Ji" OR "Taiji" OR "Taijiquan" OR "T'ai Chi" OR "Tai Chi Chuan" OR "Qi gong" OR "Ch'i Kung" OR "Yijinjing" OR "Baduanjin" OR "Wuqinxi" OR "traditional Chinese exercise")

#2 TITLE-ABS-KEY ("Parkinson's Disease" OR "Idiopathic Parkinson's Disease" OR "Lewy Body Parkinson's Disease" OR "Parkinson's Disease, Idiopathic" OR "Parkinson's Disease, Lewy Body" OR "Parkinson Disease, Idiopathic" OR "Idiopathic Parkinson Disease" OR "Lewy Body Parkinson Disease" OR "Primary Parkinsonism" OR "Parkinsonism, Primary" OR "Paralysis Agitans")

#3 TITLE-ABS-KEY ("randomized controlled trials" OR "clinical trial" OR "randomization" OR "randomized")

#4 #1 AND #2 AND #3

**Medline and Embase**

#1 (("Tai Ji":ab,ti OR "Tai-Ji":ab,ti OR "Tai Chi":ab,ti OR "Chi Tai":ab,ti OR "Tai Ji Quan":ab,ti OR "Quan Tai Ji":ab,ti OR "Ji Quan Tai":ab,ti OR "Taiji":ab,ti OR "Taijiquan":ab,ti OR "Tai Chi Chuan":ab,ti OR "Qigong":ab,ti OR "Qi Gong":ab,ti OR "Chi Kung":ab,ti OR "yijinjing":ab,ti OR "baduanjin":ab,ti OR "wuqinxi":ab,ti OR "traditional Chinese exercise":ab,ti)

#2 ("Parkinson's Disease":ab,ti OR "Idiopathic Parkinson's Disease":ab,ti OR "Lewy Body Parkinson's Disease":ab,ti OR "Parkinson's Disease, Idiopathic":ab,ti OR "Parkinson's Disease, Lewy Body":ab,ti OR "Parkinson Disease, Idiopathic":ab,ti OR "Idiopathic Parkinson Disease":ab,ti OR "Lewy Body Parkinson Disease":ab,ti OR "Primary Parkinsonism":ab,ti OR "Parkinsonism, Primary":ab,ti OR "Paralysis Agitans":ab,ti)

#3 ("randomized controlled trial":ab,ti OR "clinical trial" OR "randomization":ab,ti OR "randomized":ab,ti))

#4 #1 AND #2 AND #3

**Cochrane library**

(((Tai Ji)MeSH OR (Tai-Ji):ti,ab OR (Tai Chi):ti,ab OR (Chi Tai):ti,ab OR (Tai Ji Quan):ti,ab OR (Quan Tai Ji):ti,ab OR (Ji Quan Tai):ti,ab OR (Taiji):ti,ab OR (Taijiquan):ti,ab OR (T'ai Chi):ti,ab OR (Tai Chi Chuan):ti,ab OR (Qigong)MeSH OR (Qi Gong):ti,ab OR (Chi Kung):ti,ab OR (yijinjing):ti,ab OR (baduanjin):ti,ab OR (wuqinxi):ti,ab OR (traditional Chinese exercise):ti,ab) AND ((Parkinson's Disease):ab,ti OR (Idiopathic Parkinson's Disease):ab,ti OR (Lewy Body Parkinson's Disease):ab,ti OR (Parkinson's Disease, Idiopathic):ab,ti OR (Parkinson's Disease, Lewy Body):ab,ti OR (Parkinson Disease, Idiopathic):ab,ti OR (Idiopathic Parkinson Disease):ab,ti OR (Lewy Body Parkinson Disease):ab,ti OR (Primary Parkinsonism):ab,ti OR (Parkinsonism, Primary):ab,ti OR (Paralysis Agitans):ab,ti) AND ((randomized controlled trial):ti,ab, OR (clinical trial):ti,ab, OR (randomized):ti,ab OR (randomization):ti,ab))

**Web of Science**

((TI= (Tai Ji OR Tai-ji OR Tai Chi OR Chi Tai OR Tai Ji Quan OR Quan Tai Ji OR Ji Quan Tai OR Taiji OR Taijiquan OR T'ai Chi OR Tai Chi Chuan OR Qigong OR Qi Gong OR Chi Kung OR yijinjing OR baduanjin OR wuqinxi OR traditional Chinese exercise) OR AB= (Tai Ji OR Tai-ji OR Tai Chi OR Chi Tai OR Tai Ji Quan OR Quan Tai Ji OR Ji Quan Tai OR Taiji OR Taijiquan OR T'ai Chi OR Tai Chi Chuan OR Qigong OR Qi Gong OR Chi Kung OR yijinjing OR baduanjin OR wuqinxi OR traditional Chinese exercise)) AND (TI=(Parkinson's Disease OR Idiopathic Parkinson's Disease OR Lewy Body Parkinson's Disease OR Parkinson's Disease, Idiopathic OR Parkinson's Disease, Lewy Body OR Parkinson's Disease, Idiopathic OR Idiopathic Parkinson Disease OR Lewy Body Parkinson Disease OR Primary Parkinsonism OR Parkinsonism, Primary OR Paralysis Agitans) OR AB=(Parkinson's Disease OR Idiopathic Parkinson's Disease OR Lewy Body Parkinson's Disease OR Parkinson's Disease, Idiopathic OR Parkinson's Disease, Lewy Body OR Parkinson's Disease, Idiopathic OR Idiopathic Parkinson Disease OR Lewy Body Parkinson Disease OR Primary Parkinsonism OR Parkinsonism, Primary OR Paralysis Agitans)) AND (TI=(randomized controlled trial OR clinical trial OR randomization OR randomized) OR AB=(randomized controlled trial OR clinical trial OR randomization OR randomized)))

**Supplementary Figure 1**. Funnel plot estimating publication bias for BBS. Visual inspection indicates symmetrical distribution, and the Begg’s test was not statistically significant, suggesting an absence of publication bias (p=0.43).

**Supplementary Figure 2**. Funnel plot estimating publication bias for TUG. Visual inspection indicates unsymmetrical distribution, and the Begg’s test was not statistically significant, suggesting a presence of publication bias (p<0.05).

**Supplementary Figure 3**. Funnel plot estimating publication bias for Stride length. Visual inspection indicates symmetrical distribution, and the Begg’s test was not statistically significant, suggesting an absence of publication bias (p=0.17).

**Supplementary Figure 4**. Funnel plot estimating publication bias for Gait velocity. Visual inspection indicates symmetrical distribution, and the Begg’s test was not statistically significant, suggesting an absence of publication bias (p=0.46).

**Supplementary Figure 5**. Funnel plot estimating publication bias for Gait velocity. Visual inspection indicates symmetrical distribution, and the Begg’s test was not statistically significant, suggesting an absence of publication bias (p=0.45).

**Supplementary Figure 6**. Funnel plot for TUG using trim and fill. The TUG is SMD= −0.38, 95% CI −0.50 to −0.25.

**Supplementary Figure 7**. Effect sizes by session duration in meta-regression for BBS.

**Supplementary Figure 8**. Effect sizes by number of sessions in meta-regression for UPDRS-III.

**Supplementary Figure 9**. Effect sizes by exercise duration in meta-regression for UPDRS-III
